# Median Arcuate Ligament Syndrome as a Rare Cause of Ischemic Duodenal Ulcers: A Case Report

**DOI:** 10.7759/cureus.92370

**Published:** 2025-09-15

**Authors:** Rani Sebrechts, Sandra Müller, Patrick Lauwers, Lieselot Potums, Jeroen Hendriks

**Affiliations:** 1 Thoracic and Vascular Surgery, Antwerp University Hospital, Edegem, BEL

**Keywords:** dunbar syndrome, duodenal ulcer, median arcuate ligament release, median arcuate ligament syndrome, mesenteric ischemia

## Abstract

The median arcuate ligament syndrome is a rare cause of postprandial abdominal pain. Diagnosis is often delayed because of the heterogeneous presentation. This case report describes a patient with worsening epigastric pain and a new onset of gastrointestinal bleeding after a robot-assisted median arcuate ligament release. Diagnostic angiography showed a patent celiac artery without residual stenosis, whereas multiple ischemic and hemorrhagic duodenal ulcers were observed upon gastroscopy. After additional medical treatment with high-dose proton pump inhibitors, the patient became asymptomatic with complete healing of the ulcer. This finding is unusual, as duodenal ulcers are not typically suggestive of median arcuate ligament syndrome. Therefore, median arcuate ligament syndrome should be considered as a contributing factor in patients with therapy-resistant duodenal ulcers.

## Introduction

The median arcuate ligament syndrome (MALS), or Dunbar syndrome, is a rare condition in which the celiac artery is compressed by the median arcuate ligament. The actual prevalence is still unknown, as many physicians are not familiar with this syndrome and its varied presentation. The majority of patients are female (4:1) with a mean age of 30-50 years old [1]. The characteristic triad of symptoms consists of postprandial epigastric pain, abdominal bruit and weight loss. Other common symptoms are nausea, vomiting and food aversion [2].

Definitive diagnosis remains challenging. Recent international guidelines define MALS as the combination of imaging studies showing compression of the celiac artery by the median arcuate ligament, in concurrence with fitting symptoms. This should always be evaluated by a dedicated multidisciplinary team [3].

Duodenal ulcers are not typically associated with MALS. In current literature, this has been insufficiently researched to establish a causal relation. Only a few case reports have previously suggested a possible association [4-6]. Because of the lack of existing data, it is important to raise awareness of this possibility in order to aid with diagnostic challenges. This report describes a case where these might be related, in order to exhibit the clinical relevance of screening for MALS in patients with therapy-resistant duodenal ulcers.

## Case presentation

A 44-year-old male patient was referred to our department. He only had a history of asthma, managed by a low-dose inhalation corticosteroid (salbutamol 100mcg), which he used intermittently. He ceased smoking over 10 years ago. The patient suffered from varying abdominal symptoms for about 10 years. In 2022, extensive workup was performed, including an endocrinological and pancreatic analysis, test for coeliac disease, ultrasound imaging and cancer screening. These were all completely normal. Interestingly, a complete gastro- and colonoscopy including the duodenal bulb were performed, showing no abnormalities. He was consequently diagnosed with irritable bowel syndrome and treated accordingly.

In 2025, he returned with a progressive problem of epigastric pain 30 minutes after each meal, often accompanied by diarrhea. Over the years, he developed a severe fear of eating, resulting in a BMI of 15. There was no excessive use of painkillers, in particular of anti-inflammatory drugs. A trial with high-dose proton pump inhibitors (PPI) was unsuccessful in relieving his symptoms.

**Figure 1:**
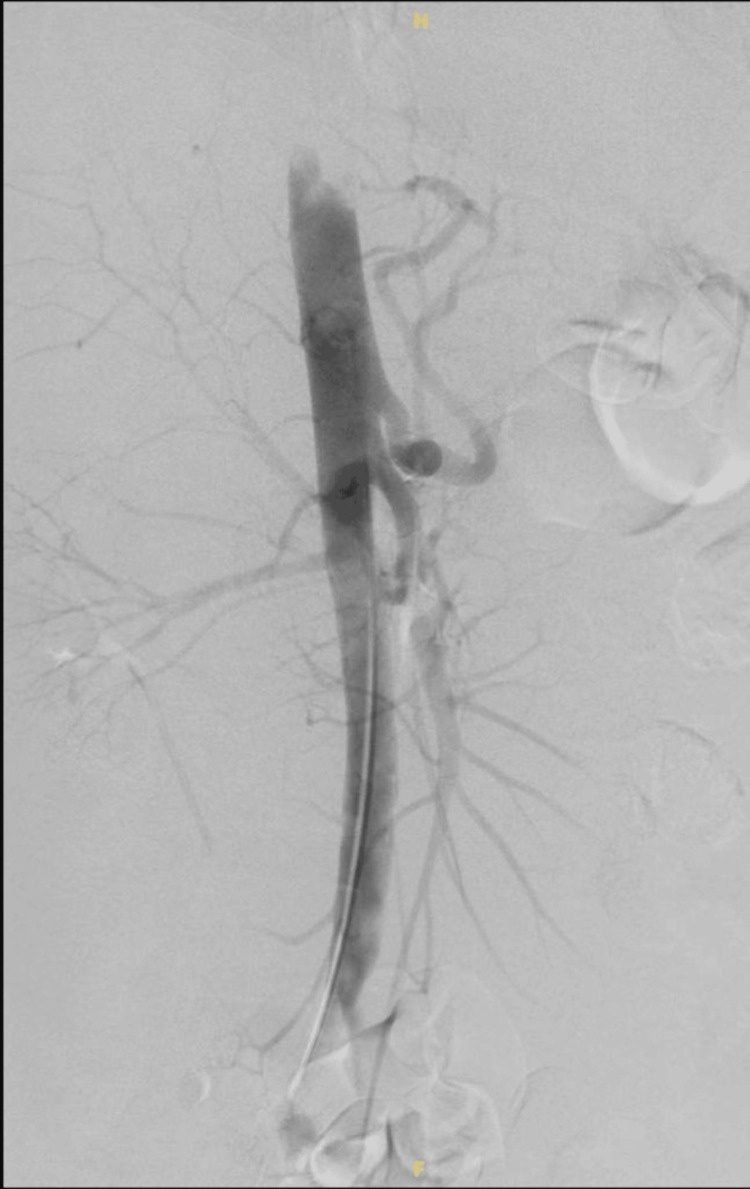
Digital subtraction angiography of the celiac artery

**Figure 2:**
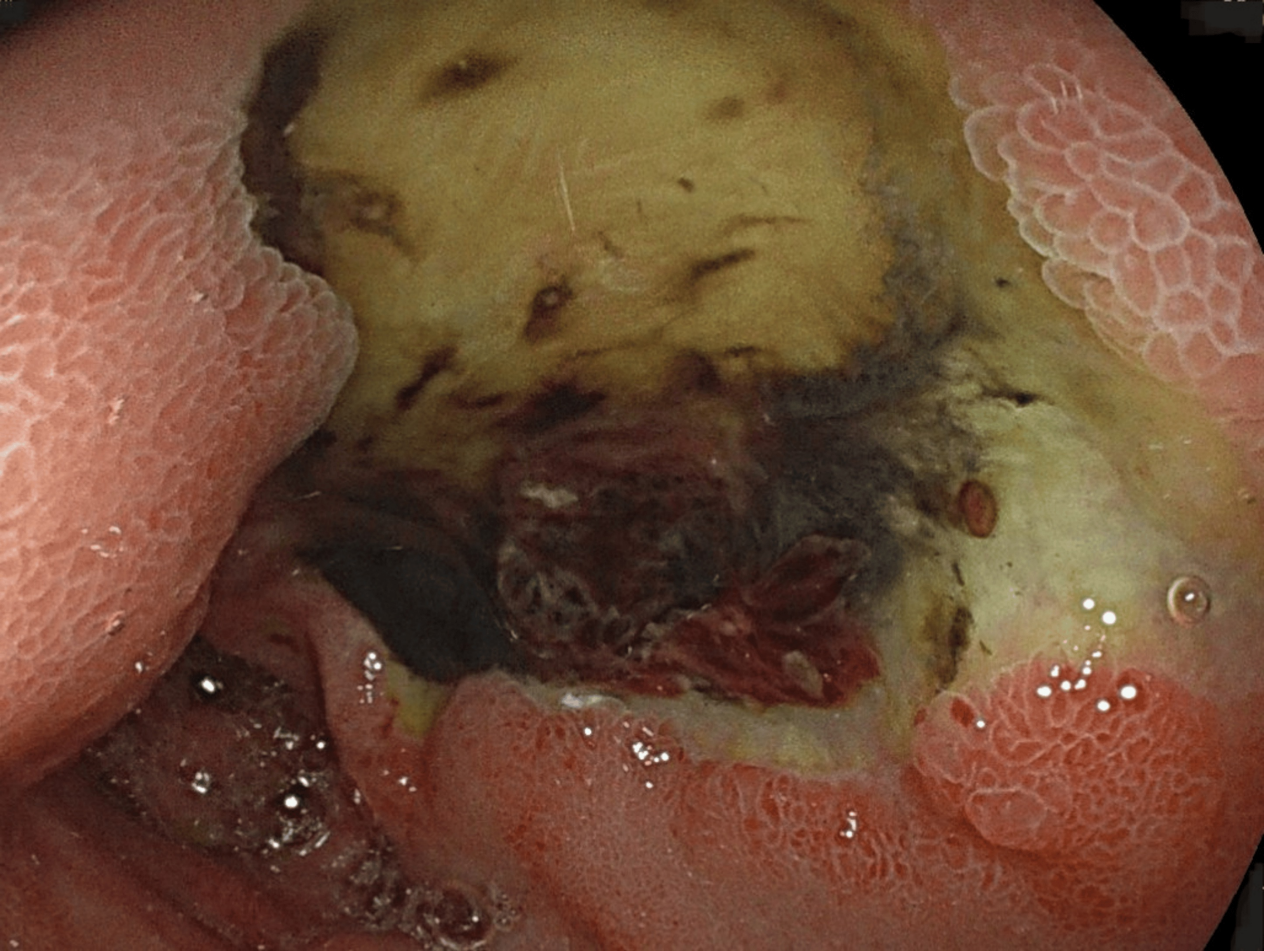
Initial gastroscopy Ischemic duodenal ulcer

Positron emission tomography (PET) scan revealed no abnormalities. Subsequent computed tomography angiography (CTA) showed a respiratory-related compression of the celiac artery. In the workup prior to referral for surgery, the patient did not get a new gastroscopy. The imaging, in combination with the severe abdominal symptoms, suggested the diagnosis of MALS. A robot-assisted median arcuate ligament release was performed. On the first postoperative day, the patient was discharged without any abdominal complaints. Prophylactic low molecular weight heparin (LMWH) was started according to protocol. As per standard of care in our department, a complementary diagnostic angiography to assess potential residual stenosis of the celiac artery was planned within five days. However, the patient was readmitted two days later with diarrhea and black stools, a general feeling of tiredness and dyspnea with exercise. The postprandial epigastric pain had returned and was even worse compared to the preoperative condition. Clinical examination was unremarkable, apart from some slight epigastric tenderness upon palpation. His vital signs remained within normal range. Standard blood analysis revealed a new microcytic anemia with a hemoglobin of 9.4 g/dl. Because of this, upper gastrointestinal bleeding was suspected. However, it was important to rule out complications at the level of the celiac artery. The scheduled diagnostic angiography was performed urgently, but showed no residual stenosis of the celiac artery (Figure 1). Extensive duodenal ulcers were observed in the duodenal bulb during gastroscopy. These were described as six giant FORREST IIb and III lesions with signs of ischemia and previous bleeding (Figure 2). Biopsy showed no signs of malignancy or Helicobacter pylori involvement. The patient was treated accordingly with high-dose PPI and iron supplements; the LMWH was discontinued and he was subsequently discharged.

**Figure 3:**
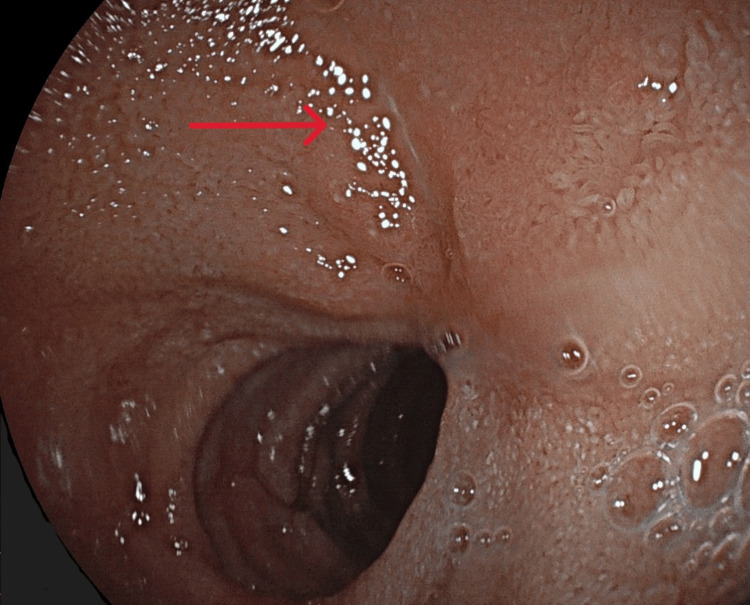
Relook gastroscopy Arrow: remaining linear erosion

At his first follow-up appointment one week later, all abdominal symptoms were resolved. Food intake increased slowly but steadily toward normal proportions. His further recovery was uneventful, with his blood count improving accordingly. Six weeks after the initial gastroscopy, a scheduled relook gastroscopy was performed. This showed nearly complete healing of the ulcers, with nothing but a linear erosion surrounded by a stellar scar remaining (Figure 3). The patient reported a significant improvement in his appetite, energy level and overall quality of life.

## Discussion

Ever since MALS was first described in 1963, it has remained a controversial diagnosis. The pathophysiology is still insufficiently understood. It is commonly associated with a certain pattern of symptoms including postprandial pain, nausea, food aversion and weight loss. Not all of these symptoms can be explained by mesenteric ischemia, as many individuals have compression of the celiac artery on CTA, without the typical symptoms. Therefore, a neuropathic component originating from the celiac plexus is considered to be involved as well [7].

The clinical diagnosis is mostly based on exclusion and pattern recognition [2]. The most recent international guidelines recommend the combination of imaging studies showing compression of the celiac artery by the median arcuate ligament, in concurrence with fitting symptoms. This should always be evaluated by a dedicated multidisciplinary team [3]. According to the European Society for Vascular Surgery (ESVS) guidelines, duplex ultrasound should always be performed first as it is non-invasive and has a high degree of sensitivity and specificity. This is usually complemented by CTA. All imaging studies should be performed in inspiration and expiration [8]. Our patient in fact had a significant increase in celiac artery compression upon expiration.

Surgical treatment of MALS remains controversial. Care should be taken not to treat a patient solely based on radiological findings. In current guidelines, there is no clear standard of treatment. However, minimally invasive median arcuate ligament release is most commonly suggested [3]. This can be done either by a laparoscopic or a robot-assisted approach. Both techniques are deemed safe and effective in resolving both the vascular and neurogenic compression, resulting in a high degree of symptom relief [9,10]. Endovascular therapy, consisting of celiac artery stenting without surgical release, has proven ineffective. In case of residual stenosis however, it can be a useful addition [1]. Therefore, in our center a routine diagnostic angiography with stenting as needed is performed after each surgical release. 

Duodenal ulcers are rarely described as being associated with MALS. In this case, the extensive ulcers can be insufficiently explained without the involvement of MALS. The patient did use inhalation corticosteroids, but only sporadically. Other contributing factors were the postoperative heparin use and surgical stress. However, since these events happened only 48 hours after surgery, it is highly unlikely that there were no ulcers before the surgery. Furthermore, there was no Helicobacter pylori involvement. 

Only a few case reports have previously suggested a possible association between the vascular compression, postprandial pain and duodenal ulcers [4-6]. These cases had a similar course to this patient, where the symptoms were only controlled when both the ulcers and the MALS were addressed. Early recognition of this association might have significant clinical relevance in patients with PPI-resistant ulcers, as it offers an additional treatment option that is not immediately expected. Therefore, this report aims to raise awareness surrounding MALS and the simultaneous occurrence of duodenal ulcers.

## Conclusions

In selected cases, MALS could be an underestimated contributing factor in the formation of duodenal ulcers. Patients with suggestive clinical presentation in combination with therapy resistant ulcers should be screened for MALS involvement. Care should be taken when interpreting these results since celiac artery compression is often a radiological finding without clinical repercussions.

When MALS is suspected in patients with duodenal ulcers refractory to medical therapy, surgical release of the median arcuate ligament should be considered as an adjunct intervention to achieve optimal symptom control and therapeutic outcome.

## References

[1] Kim EN, Lamb K, Relles D, Moudgill N, DiMuzio PJ, Eisenberg JA (2016). Median arcuate ligament syndrome-review of this rare disease. JAMA Surg.

[2] Goodall R, Langridge B, Onida S, Ellis M, Lane T, Davies AH (2020). Median arcuate ligament syndrome. J Vasc Surg.

[3] Terlouw LG, Moelker A, Abrahamsen J (2020). European guidelines on chronic mesenteric ischaemia - joint United European Gastroenterology, European Association for Gastroenterology, Endoscopy and Nutrition, European Society of Gastrointestinal and Abdominal Radiology, Netherlands Association of Hepatogastroenterologists, Hellenic Society of Gastroenterology, Cardiovascular and Interventional Radiological Society of Europe, and Dutch Mesenteric Ischemia Study group clinical guidelines on the diagnosis and treatment of patients with chronic mesenteric ischaemia. United European Gastroenterol J.

[4] Sunkara T, Caughey ME, Zhen KC, Chiong B, Gaduputi V (2017). Dunbar syndrome-a rare cause of foregut ischemia. J Clin Diagn Res.

[5] Bronze SM, Conceição D, Mendes M, Cardoso F, Torres D, Coimbra E, Bilhim T (2024). Refractory duodenal ulcer in a patient with median arcuate ligament compression: a treatment challenge. ACG Case Rep J.

[6] Gunduz Y, Asil K, Aksoy YE, Tatlı Ayhan L (2014). Clinical and radiologic review of uncommon cause of profound iron deficiency anemia: median arcuate ligament syndrome. Korean J Radiol.

[7] Chaum M, Shouhed D, Kim S, Walts AE, Marchevsky AM (2021). Clinico-pathologic findings in patients with median arcuate ligament syndrome (celiac artery compression syndrome). Ann Diagn Pathol.

[8] Koelemay MJ, Geelkerken RH, Kärkkäinen J (2025). Editor's Choice - European Society for Vascular Surgery (ESVS) 2025 clinical practice guidelines on the management of diseases of the mesenteric and renal arteries and veins. Eur J Vasc Endovasc Surg.

[9] Do MV, Smith TA, Bazan HA, Sternbergh WC 3rd, Abbas AE, Richardson WS (2013). Laparoscopic versus robot-assisted surgery for median arcuate ligament syndrome. Surg Endosc.

[10] Metz FM, Blauw JT, Brusse-Keizer M, Kolkman JJ, Bruno MJ, Geelkerken RH (2022). Systematic review of the efficacy of treatment for median arcuate ligament syndrome. Eur J Vasc Endovasc Surg.

